# Analytical solutions for the Klein–Gordon equation with combined exponential type and ring-shaped potentials

**DOI:** 10.1038/s41598-024-53650-8

**Published:** 2024-03-06

**Authors:** A. I. Ahmadov, Sh. M. Nagiyev, A. N. Ikot, V. A. Tarverdiyeva

**Affiliations:** 1 Department of Theoretical Physics, Baku State University, Z. Khalilov St. 23, 1148 Baku, Azerbaijan; 2 Institute for Physical Problems, Baku State University, Z. Khalilov St. 23, 1148 Baku, Azerbaijan; 3 Institute of Physics, Ministry of Science and Education, H. Javid Avenue, 131, 1143 Baku, Azerbaijan; 4 Theoretical Physics Group, Department of Physics, University of Port Harcours, P M B 5323, Choba, Port Harcourt, Nigeria

**Keywords:** Physics, Quantum physics

## Abstract

In this study, we have successfully obtained the analytical solutions for the Klein–Gordon equation with new proposed a non-central exponential potential $V\left( r \right) = D\left[ {1 - \sigma_{0} \coth (\alpha r)} \right]^{2} + (\eta_{1} + \eta_{2} \cos \theta )/r^{2} \sin^{2} \theta$. Our approach involves a proper approximation of the centrifugal term, with ${l}{\prime}$ representing the generalized orbital angular momentum quantum number, and the utilization of the Nikiforov–Uvarov method. The resulting radial and angular wave functions are expressed in terms of Jacobi polynomials, and the corresponding energy equation is also derived. Our calculations of the eigenvalues for arbitrary quantum numbers demonstrated significant sensitivity to potential parameters and quantum numbers. Additionally, we evaluate the dependence of energy eigenvalues on screening parameter $\alpha$ for arbitrary quantum numbers $n_{r}$ and $N$ to establish the accuracy of our findings. Furthermore, we determine the non-relativistic limits of the radial wave function and energy equation, which align with corresponding previous results in the case where $\eta_{1} = \eta_{2} = 0$.

## Introduction

One of the main tasks of relativistic and nonrelativistic quantum mechanics is the description of the motion of microparticles (molecules, atoms, atomic nuclei, etc.) in an external potential field^1–5^. In this case, as is known, depending on the type of potential energy of particle interaction with the field $V(\mathbf{r})$, the equation of motion can have solutions that belong to both discrete and continuous energy spectra. The solutions corresponding to the discrete spectrum describe the bound states of the particle, the states of the continuous spectrum describe the scattering of the particle in the potential field. Bound states correspond to the finite motion of a particle, when the particle is held by the forces of attraction of the field in a limited region of space. In other words, bound states arise when the interaction potential has the form of a well. If the height of the potential well is ${V}_{0}$, then at particle energies $E<{V}_{0}$ the energy spectrum is discrete, and at energies $E>{V}_{0}$ it is continuous. Continuous states correspond to the infinite motion of a particle, when the particle comes from infinity and, scattering, goes to infinity. We emphasize that when a particle is in a potential field, in addition to bound states and scattering states, there can also be unstable states that decay with time. Indeed, if the energy of the particle is greater than the value of the potential at infinity ${V}_{\infty }$, i.e. belongs to the region ${V}_{\infty }<E<{V}_{0}$, then sooner or later, due to the tunnel effect, the particle will fly out of the region of attraction of potential forces.

The basic equation of motion of nonrelativistic quantum mechanics is the Schrödinger equation. Based on this equation, nonrelativistic quantum mechanics explains the main properties of molecular, atomic and nuclear physicochemical phenomena^1,2,5^. Nonrelativistic quantum mechanics underlies the theory of solids. In all these cases, different phenomenological interaction potentials are used. The synthesis of non-relativistic quantum mechanics with the theory of relativity led to the creation of relativistic quantum mechanics and quantum field theory. If the energy of a particle is comparable to its rest energy, but the interaction potential is still insufficient for the formation of particle-antiparticle pairs, then the motion of such a particle must be described by relativistic quantum mechanics. The basic equations of motion of relativistic quantum mechanics are the Klein-Gordon (KG) and Dirac equations^3–5^. In this regard, we also note that there is another version, namely the finite-difference version of relativistic quantum mechanics, which was developed and applied to solve a number of problems in particle physics in papers (see^6^ and the references therein). An analogue of the Schrödinger equation in finite-difference relativistic quantum mechanics is a Schrödinger-type finite-difference equation in configuration space. Finite-difference relativistic quantum mechanics has many important features of non-relativistic quantum mechanics. In particular, the wave functions here admit a probabilistic interpretation.

In the framework of relativistic and nonrelativistic quantum mechanics, potential models have always played an important role and will play, perhaps, even the main role in the physics of the nucleus and elementary particles, in the physics of atoms and molecules. The importance of a potential model is primarily determined by how well it describes certain properties of the physical system under study. The physical properties of micro-objects in potential models are described and interpreted using various wave equations, such as the Schrödinger equation, the Dirac equation, the KG equation and the relativistic finite difference equation. In this case, exact or phenomenologically introduced central and non-central interaction potentials are widely used. There are many such potentials. The most well-known potentials widely used in the relativistic and nonrelativistic energy regions are the Coulomb potential and the harmonic oscillator potential, as well as their various combinations. The list of some other phenomenological potentials of interaction can found in Refs.^7–17^.

There are many works^17–49^ in which the problems of both non-relativistic^17–35^ and relativistic^36–51^ bound states and scattering states are studied. We also note that the relativistic KG and Dirac wave equations for a charge in an external electromagnetic field form the basis of quantum electrodynamics^1^. We emphasize that the class of noncentral potentials^6,14–38^ plays a particularly important role in quantum mechanics, nuclear physics, and theoretical chemistry, which can be represented as

1 $$V\left( {\mathbf{r}} \right) = V\left( r \right) + \frac{{f\left( {\uptheta ,\upvarphi } \right)}}{{r^{2} }},$$

where $V\left(r\right)$ is some central (for example, Coulomb^15^ or oscillatory^17^) potential, and $f(\uptheta ,{ \upvarphi })$ is some function of angles characterizing the annularity of the potential. Non-central potentials were apparently considered for the first time in the framework of nonrelativistic quantum mechanics in^14–17^. These models allow the separation of variables in the Schrödinger equation in several coordinate systems, and a dynamic invariance algebra has been constructed for them^17–19^.

Non-central potentials are good models in quantum chemistry, in nuclear and atomic physics, and in molecular physics. Ring potentials can be used in quantum chemistry to describe organic ring-shaped molecules such as benzene, and in nuclear physics to study the interactions of a deformed pair of nuclei and spin–orbit coupling when particles move in potential fields. This potential is also used as a mathematical model for describing the vibrations of diatomic molecules and is a convenient model in other physical situations. For example, the Hartmann potential^15^ is one of the useful combinations in the form of Eq. (1), which is used to describe organic molecules in the framework of nonrelativistic quantum mechanics. Other important off-center potentials were proposed by Hautot^16^ to describe the motion of a charged non-relativistic particle in an electric field. The Hartmann potential is a special case of one of the Hautot potentials. In^17^, Quesne investigated a new non-central potential, obtained by replacing the Coulomb part of the Hartmann potential by a harmonic oscillator term. In^51^, the relativistic problem of the motion of a scalar particle in a non-central field, which is equal to the sum of the Coulomb and one of the Hautot potentials, was solved. Here also a dynamic symmetry group for the radial wave functions is constructed.

On the other hand, within the framework of nonrelativistic quantum mechanics, the authors of^52^ found the energy eigenvalues for a hyperbolic potential of the form^53^ (also called the exponential type potential)

2 $$V\left( r \right) = D\left[ {1 - \sigma_{0} \coth \left( {\upalpha {\text{r}}} \right)} \right]^{2} ,$$

where $D$, ${\sigma }_{0} {\text{and}} \alpha$ are positive constant parameters. It was shown in^52–54^ that this potential is closely related to the Morse, Kratzer, Coulomb potentials, the harmonic oscillator, and other interaction potentials. The hyperbolic potential is an important exponential type potential^53^. For more information about its properties and possible applications, the reader can refer to^52–54^.

As well known that an analytical and exact solution, for example, of the KG equation is possible only in the s-wave case with an orbital quantum number $l = 0$ for some potentials. In this regard, we emphasize that, as is known, one of the frequently used approximate methods for solving equations for any $l \ne 0$ value of orbital quantum number of motion with a central or noncentral potential are the Greene and Aldrich and Pekeris approximations^55,56^. These approximations widely applied to the solution of the KG equation for different potentials, such as Hulthén potential^57,58^, generalized Hulthén potential^59–61^, Manning–Rosen potential^62,63^, Wood–Saxon potential^64^, generalized hyperbolic potential^65^, Deng-Fan molecular potentials^66^, inversely quadratic Hellman potential^67^ and Kratzer Potential^68^ and similarly for the case of combined potentials like Hulthén plus Yukawa potential^69–71^, Manning–Rosen plus a class of Yukawa^72^, Hellmann plus modified Kratzer potential^73^, Mobius squared plus Eckart potential^74^, and Eckart plus a ring-shaped like potential studied in Refs.^75^.

The purpose of this work is to analytically solve the KG equation for the new proposed as sum of hyperbolic potential and a ring-shaped potential of the form

3 $$V_{{{\text{rsh}}}} \left( {r,\theta } \right) = \frac{{\eta_{1} + \eta_{2} \cos\uptheta }}{{{\text{r}}^{2} \sin^{2} \theta }} \equiv \frac{{f\left(\uptheta \right)}}{{{\text{r}}^{2} }},$$

where $\eta_{1}$ and $\eta_{2}$ are positive constant parameters that characterize the properties of interaction potentials. The combined potential considered in this paper is obtained as a linear combination of an exponential potential with a ring-shaped potential in the form:

4 $$V\left( {r,\theta } \right) = D\left[ {1 - \sigma_{0} \coth \left( {\upalpha {\text{r}}} \right)} \right]^{2} + \frac{{\eta_{1} + \eta_{2} \cos\uptheta }}{{{\text{r}}^{2} \sin^{2} \theta }}.$$

Here we generalize the results of^52^: firstly, to the relativistic case, and secondly, to the case of noncentral potential (4).

Let us note that some authors^41–43,45–48^ have assumed that in the KG equation the scalar potential is equal to the vector potential and have solved exactly or approximately the KG equation. In this paper, we also assume that the scalar and vector potentials are equal to each other, and we solve the KG equation, and present the approximately analytical expressions for the wave functions and for the discrete energy spectrum.

Thus, new potential (4) as self-behavior is a non-central potential.

In this study, we try to study analytical solutions of the KG equation for any value of the orbital quantum numbers $l \ne 0$ in the framework of the Greene and Aldrich approximation^55^ for the new proposed combined potential (4) using the Nikiforov-Uvarov (NU) method^76^. This method is based on solving a second-order linear differential equation by reducing it to a generalized hypergeometric type equation, which is a homogeneous second-order differential equation with polynomial coefficients of degree not exceeding the corresponding order of differentiation. Many studies show the strength and simplicity of the NU method in solving central and non-central potentials for arbitrary $l \ne 0$ states.

We emphasize that in^77^ the relativistic problem of bound states for the hyperbolic potential (2) was also considered. However, there are two differences from our work: firstly, we consider the case of a non-central potential, and secondly, we use a different approximation for the centrifugal term.

This paper is organized as follows. In Section "Brief summary of the Nikiforov–Uvarov method" given a brief summary about the Nikiforov-Uvarov method. In Section "Klein–Gordon equation for non-central potential" separates the variables in the KG equation. In Section "Radial Klein–Gordon equation" we find analytically the bound-states solutions of the radial KG equation with the non-central potential (4). In Section "Angular part of the Klein–Gordon equation", we present the solution of the angular part of the KG equation. We discuss the nonrelativistic limits of the radial wave function and the energy equation and some of their particular cases in Section "Limiting case". We present numerical estimates for the energy eigenvalues and their corresponding normalized wave functions in Section "Discussion of the numerical results". Finally, we conclude our work in Section "Conclusion".

## Brief summary of the Nikiforov–Uvarov method

The NU method solves a second-order differential equation of the following form^76^:

5 $$\chi^{\prime\prime}(g) + \frac{{\tilde{\tau }(g)}}{\sigma (g)}\chi^{\prime}(g) + \frac{{\tilde{\sigma }(g)}}{{\sigma^{2} (g)}}\chi (g) = 0.$$

Here $\sigma (g),$ and $\tilde{\sigma }(g)$ are polynomials of degree at most 2, a $\tilde{\tau }(g)$ is a is a polynomial of degree at most 1. Then, using the following factorization for the function $\chi \left( s \right)$:

6 $$\chi \left( g \right) = \phi (g)y(g),$$

we reduce Eq. (5) to a hypergeometric type equation of the form

7 $$\sigma (g)y^{\prime\prime}(g) + \tau (g)y^{\prime}(g) + \lambda y(g) = 0,\quad \lambda = {\text{const}}.$$

Function $\varphi (g)$ must satisfy the logarithmic derivative

8 $$\frac{{\phi^{\prime}(g)}}{\phi (g)} = \frac{\pi (g)}{{\sigma (g)}}$$

with

9 $$\pi (g) = \frac{{\sigma^{\prime}(g) - \tilde{\tau }(g)}}{2} \pm \sqrt {\left( {\frac{{\sigma^{\prime}(g) - \tilde{\tau }(g)}}{2}} \right)^{2} - \tilde{\sigma }(g) + k\sigma (g)} ,$$

where the primes denote the derivative with respect to $g$, and it can be at most first order. The term inside the square root is transposed as the zero discriminant of the second-order polynomial. Therefore, the equation reduces to an equation of hypergeometric type, where one of its solutions is $y\left( g \right)$. Therefore, the expression for $k$ is found after solving such an equation by the NU method.

Therefore, the equation reduces to an equation of hypergeometric type, where one of its solutions is $y\left( g \right)$. Therefore, the polynomial expression $\overline{\sigma }(g) = \tilde{\sigma }(g) + \pi^{2} (g) + \pi (g)\left[ {\tilde{\tau }(g) - \sigma^{\prime}(g)} \right] + \pi^{\prime}(g)\sigma (g)$ can be divided by a factor $\sigma (g)$, such that $\overline{\sigma }(g)/\sigma (g) = \lambda$. Here we use the following relations

10 $$\overline{\lambda } = k + \pi^{\prime}(g),$$

11 $$\tau (g) = \tilde{\tau }(g) + 2\pi (g),$$

where $\tau (g)$ satisfy the condition ${\tau }{\prime}\left(g\right)<0.$ Here $k$ is parameter, the definition of which is essential in the calculation $\pi \left( g \right)$. This parameter is simply determined from expression (9) by setting the square root discriminant to zero. Hence the general quadratic equation for $k$ can be obtained. Then $k$ can be used to calculate the energy eigenvalues using the formula

12 $$\overline{\lambda } = k + \pi^{\prime}(g) = - n\tau^{\prime}(g) - \frac{n(n - 1)}{2}\sigma^{\prime\prime}(g),\quad n = 0,1,2, \ldots$$

Polynomial solutions $y\left( g \right)$ are determined by the Rodrigues formula:

13 $$y_{n} (g) = \frac{{C_{n} }}{\rho (g)}\frac{{d^{n} }}{{dg^{n} }}\left[ {\sigma^{n} (g)\rho (g)} \right],$$

where $C_{n}$ normalization constant, and $\rho (g)$ denotes a weight function that obeys [81],

14 $$\frac{d}{dg}[\sigma (g)\rho (g)] = \tau (g)\rho (g).$$

Usually this is called Pearson's differential equation.

## Klein–Gordon equation for non-central potential

We know well that in relativistic quantum mechanics the dynamics of spin-zero particles is described by the KG equation. For scalar $V_{1} ({\mathbf{r}})$ and vector $V_{2} ({\mathbf{r}})$ potentials, the KG equation in atomic units $(\hbar = c = 1)$ in a spherical coordinate system has the form^3^

15 $$[ - \nabla^{2} + (M + V_{1} ({\mathbf{r}}))^{2} ]\psi ({\mathbf{r}}) = [E - V_{2} ({\mathbf{r}})]^{2} \psi ({\mathbf{r}}),$$

where *M* and *E* are the mass and energy of the relativistic particle. For simplicity, we assume that $V_{1} ({\mathbf{r}})$ and $V_{2} ({\mathbf{r}})$ are equal:

16 $$V_{1} ({\mathbf{r}}) = V_{2} ({\mathbf{r}}) = V({\mathbf{r}}).$$

Replacement (16) in Eq. (15) gives

17 $$[\nabla^{2} + \varepsilon^{2} - \gamma V({\mathbf{r}})]\,\psi ({\mathbf{r}}) = 0,\quad \varepsilon^{2} = E^{2} - M^{2} ,\quad \gamma = 2(E + M).$$

where, as we know,

18 $$\nabla^{2} = \frac{1}{{r^{2} }}\partial_{r} (r^{2} \partial_{r} ) + \frac{{\Delta_{\theta ,\phi } }}{{r^{2} }},\quad \Delta_{\theta ,\phi } = \frac{1}{\sin \theta }\partial_{\theta } (\sin \theta \,\partial_{\theta } ) + \frac{1}{{\sin^{2} \theta }}\partial_{\phi }^{2} .$$

Taking into account (3) and (4), Eq. (17) can be written as

19 $$\left[ {\nabla^{2} + \varepsilon^{2} - \gamma \,V(r) - \gamma \frac{f(\theta )}{{r^{2} }}} \right]\,\psi ({\mathbf{r}}) = 0.$$

This equation in spherical coordinates admits separation of variables. Therefore, the wave function $\psi ({\mathbf{r}})$ can be written in the factorized form:

20 $$\psi ({\mathbf{r}}) = \frac{\chi (r)}{r}\Theta (\theta )\Phi (\phi ).$$

It should be immediately noted that, since the operator $\hat{L} = - i\partial_{\phi }$ commutes with the Hamiltonian of Eq. (19), the function $\Phi (\phi )$ has the standard form

21 $$\Phi (\phi ) \equiv \Phi_{m} (\phi ) = \frac{1}{{\sqrt {2\pi } }}e^{im\phi } ,\quad m = 0,\, \pm 1,\, \pm 2, \ldots$$

After substituting function (20) into Eq. (19), the following system of second-order differential equations is obtained:

22 $$\chi^{\prime\prime}(r) + \left[ {\varepsilon^{2} - \gamma \,V(r) - \frac{\mu }{{r^{2} }}} \right]\chi (r) = 0,$$

23 $$\Theta^{\prime \prime } (\theta ) + ctg\theta \cdot \Theta^{\prime } (\theta ) + \left[ {\mu - \frac{{m^{2} + \gamma (\eta_{1} + \eta_{2} \cos \theta )}}{{\sin^{2} \theta }}} \right]\Theta (\theta ) = 0,$$

where $\mu$ and *m* are separation constants. Let's define the generalized orbital quantum number ${l}{\prime}$, which in the general case is not an integer: $\mu ={l}{\prime}\left({l}{\prime}+1\right)$. It dependents on the energy, т.e. ${l}{\prime}={l}{\prime}(E)$. In the nonrelativistic limit, this dependence is vanish. Obviously, in the absence of a ring-shaped potential, the number ${l}{\prime}$ will coincide with the orbital quantum number $l$ =0, 1, 2, …, i.e. at $\eta_{1} = \eta_{2} = 0$ we have

24 $$l^{\prime} = l\quad and\quad \Theta (\theta ) = P_{l}^{m} \left( {\cos \uptheta } \right) ,$$

where ${P}_{l}^{m}\left(\mathrm{cos\theta }\right)$ is Legendre function.

From Eq. (22) we can extract the effective potential in the following form:

25 $$V_{eff} (r) = \gamma \,D\,(1 - \sigma_{0} \coth (\alpha r))^{2} + \frac{\mu }{{r^{2} }}.$$

**Figure 1:**
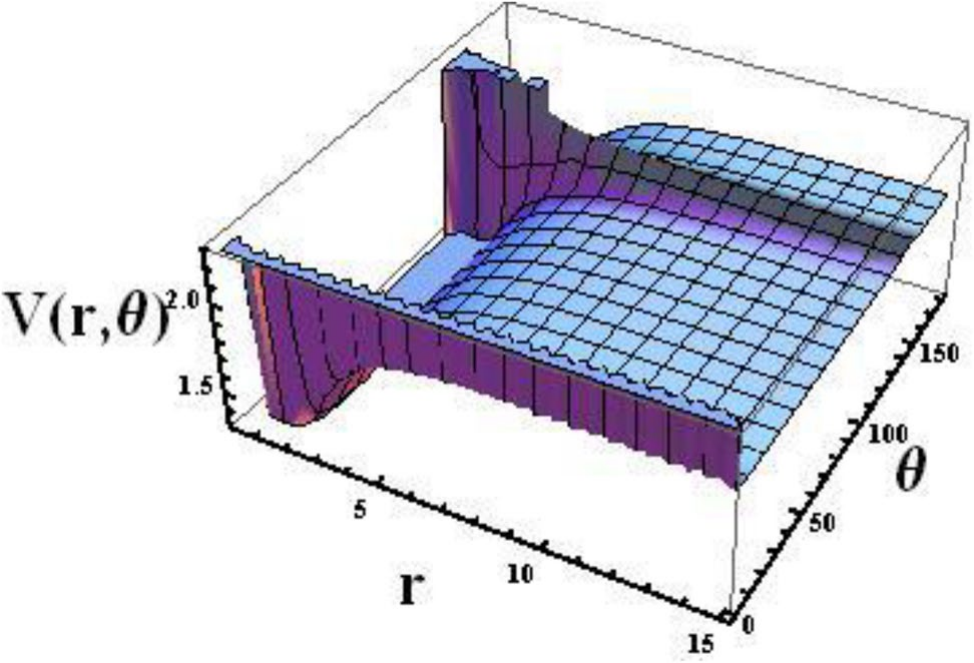
3D representation of the novel combined potential with respect separation distance $r$ and angle $\theta$. Here we take the potential parameters as $D = 3$, $\sigma_{0} = 0.20$, $\alpha = 0.30$, $\eta_{1} = 0.01$ and $\eta_{2} = 0.002$.

**Figure 2:**
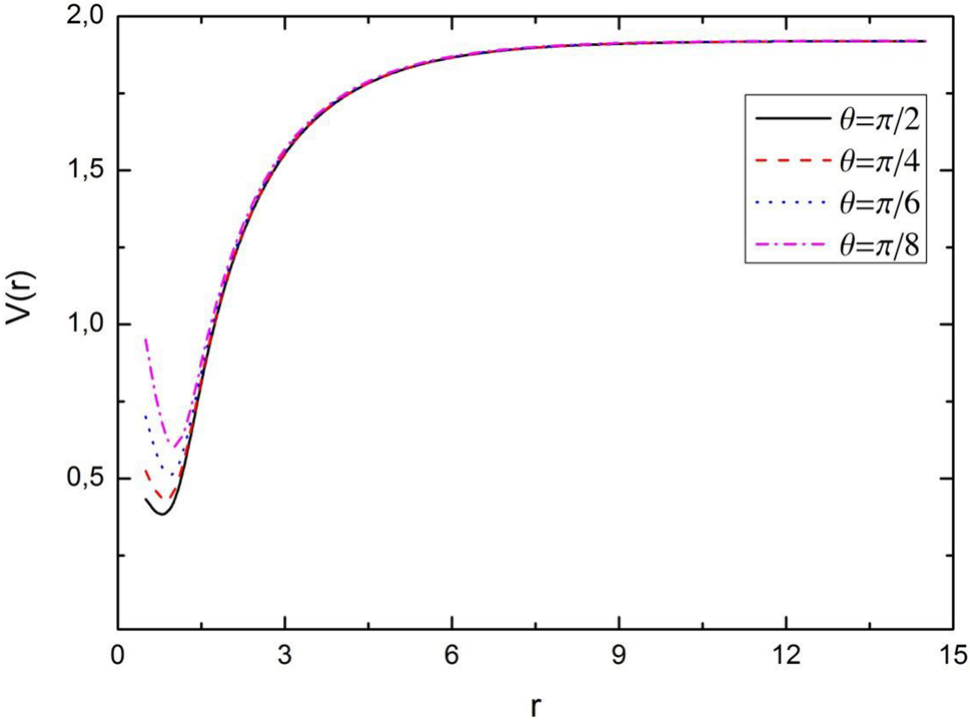
Graphical representation of the novel combined potential with respect separation distance $r$. Here we take the potential parameters as $D = 3$, $\sigma_{0} = 0.20$, $\alpha = 0.30$, $\eta_{1} = 0.01$ and $\eta_{2} = 0.002$.

In Fig. 1 shows the dependences of potential (4) $V\left(r,\theta \right)$ both on the separation distance $r$ and on the angle $\theta$. Also in Fig. 2, we visualized the dependence of the potential as a function of the separation distance for some fixed values of $\theta$.

## Radial Klein–Gordon equation

In this section, applying the NU method, we find an analytical solution of the radial KG Eq. (12). The NU method is one of the effective methods for solving a certain type of second-order differential equations and is widely used to explicitly obtain the polynomial parts of the wave functions and discrete bound-state energy levels for quantum systems for some solvable interaction potentials. This has been shown by many studies, as noted above.

Let us rewrite the differential Eq. (22) in expanded form:

26 $$\chi^{\prime\prime}(r) + \left[ {\varepsilon^{2} - \gamma \,D(1 - \sigma_{0} \coth (\alpha r))^{2} - \frac{\mu }{{r^{2} }}} \right]\chi (r) = 0.$$

As can be seen, the effective potential contains the centrifugal term. Therefore, the KG equation with this potential cannot be solved analytically exactly for $\mu \ne 0$. To solve analytically Eq. (25) for the case $\mu \ne 0$, we apply a special approximation to the centrifugal potential. If $\alpha r \ll \;1$ we use the following scheme for approaching the centrifugal potential^56^:

27 $$\frac{1}{{r^{2} }} \approx \frac{{4\alpha^{2} e^{ - 2\alpha r} }}{{(1 - e^{ - 2\alpha r} )^{2} }} = \frac{{\alpha^{2} }}{{\sinh \left( {\alpha r} \right)}}.$$

After applying approximation (27) in Eq. (26), we get

28 $$\chi^{\prime\prime}(r) + \left[ {\varepsilon^{2} - \gamma \,D\left( {1 - \sigma_{0} \frac{{1 + e^{ - 2\alpha r} }}{{1 - e^{ - 2\alpha r} }}} \right)^{2} - \frac{{4\alpha^{2} \mu \,e^{ - 2\alpha r} }}{{(1 - e^{ - 2\alpha r} )^{2} }}} \right]\chi (r) = 0.$$

We will find an analytical solution of the radial Eq. (28) using the NU method.

To apply the NU method to the solution of Eq. (28), we introduce a new variable in the form $g = e^{ - 2\alpha r} \in [0,1]$, and $r \in [0,\infty )$. Then we can represent Eq. (28) in the following form:

29 $$\chi^{\prime\prime}(g) + \frac{1}{g}\chi^{\prime}(g) + \left[ {\frac{{\varepsilon^{2} }}{{4\alpha^{2} g^{2} }} - \frac{\gamma D}{{4\alpha^{2} g^{2} }}(1 - \sigma_{0} \frac{1 + g}{{1 - g}})^{2} - \mu \frac{g}{{g^{2} (1 - g)^{2} }}} \right]\chi (r) = 0,$$

or

30 $$\chi^{\prime\prime}(g) + \frac{1}{g}\chi^{\prime}(g) + \frac{1}{{g^{2} (1 - g)^{2} }}\left[ {\frac{{\varepsilon^{2} }}{{4\alpha^{2} }}(1 - g)^{2} - \frac{\gamma D}{{4\alpha^{2} }}\left[ {(1 - g)^{2} - 2\sigma_{0} (1 - g^{2} ) + (1 + g)^{2} \sigma_{0}^{2} } \right] - \mu \,g} \right]\chi (g) = 0.$$

We are interested in bound states: $E > M$, $\varepsilon > 0$. As can be seen, Eq. (30) is a suitable type for the NU method. Now we can successfully apply this method to determine the energy spectrum. Comparing Eq. (30) with Eq. (5) for polynomials $\sigma (g)$, $\tilde{\tau }(g)$ and $\tilde{\sigma }(g)$, we obtain:

31 $$\tilde{\tau }(g) = 1 - g,\quad \sigma (g) = g(1 - g),\quad \tilde{\sigma }(g) = \left( {\frac{1}{4} - a_{1} } \right)g^{2} + a_{2} g - a_{3} ,$$

where

32 $$\begin{aligned} a_{1} & = \frac{1}{4} - \frac{{\varepsilon^{2} }}{{4\alpha^{2} }} + \frac{\gamma D}{{4\alpha^{2} }}(1 + \sigma_{0} )^{2} , \\ a{}_{2} & = - \frac{{\varepsilon^{2} }}{{2\alpha^{2} }} + \frac{\gamma D}{{2\alpha^{2} }}(1 - \sigma_{0}^{2} ) - \mu , \\ a_{3} & = - \frac{{\varepsilon^{2} }}{{4\alpha^{2} }} + \frac{\gamma D}{{4\alpha^{2} }}(1 - \sigma_{0} )^{2} . \\ \end{aligned}$$

By inserting the factorization (6) in Eq. (30), we obtain a hypergeometric type equation similar to Eq. (7). Given condition (8) for $\varphi (g)$, the function $\pi (g)$ can be determined from formula (9):

33 $$\pi (g) = - \frac{g}{2} \pm \sqrt {g^{2} (a_{1} - k) - g(a_{2} - k) + a_{3} } .$$

To find the parameter $k$, we use the condition that the discriminant of the radical square trinomial in (32) is equal to zero. From here we get

34 $$k = (a_{2} - 2a_{3} ) \pm 2\sqrt {a_{3}^{2} + a_{3} (a_{1} - a_{2} )} .$$

After substituting expression (34) in Eq. (33), we obtain the following expressions for $\pi (g)$

35 $$\pi (g) = - \frac{g}{2} \pm \left\{ {\begin{array}{*{20}c} {(\sqrt {a_{3} } - \sqrt {a_{3} + a_{1} - a_{2} } )g - \sqrt {a_{3} } ,} & {k_{1} = (a_{2} - 2a_{3} ) + 2\sqrt {a_{3}^{2} + a_{3} (a_{1} - a_{2} )} ,} \\ {(\sqrt {a_{3} } + \sqrt {a{}_{3} + a_{1} - a_{2} } )g - \sqrt {a_{3} } ,} & {k_{1} = (a_{2} - 2a_{3} ) - 2\sqrt {a_{3}^{2} + a_{3} (a_{1} - a_{2} )} .} \\ \end{array} } \right.$$

Of the four possible expressions for $\pi (g)$, we choose the case when the function $\tau (g)$ has a negative derivative, since other forms are physically unacceptable. Thus, we choose the appropriate function for $\pi (g)$ and $\tau (g)$ as follows:

36 $$\pi (g) = \sqrt {a_{3} } - \left( {\sqrt {a_{3} } + \sqrt {a_{3} + a_{1} - a_{2} } + \frac{1}{2}} \right)g,$$

37 $$\tau (g) = 2\sqrt {a_{3} } - 2(\sqrt {a_{3} + a_{1} - a_{2} } + 1)g + 1$$

for $k \equiv k_{2} = (a_{2} - 2a_{3} ) - 2\sqrt {a_{3}^{2} + a_{3} (a_{1} - a_{2} )}$. Applying formula $\lambda = k + \pi^{\prime}(g)$ from (10), the parameter $\lambda$ can be written as

38 $$\lambda = (a_{2} - 2a_{3} ) - 2\sqrt {a_{3}^{2} + a_{3} (a_{1} - a_{2} )} - \frac{1}{2} - (\sqrt {a_{3} } + \sqrt {a_{3} + a_{1} - a_{2} } )$$

where

$$\begin{gathered} a_{2} - 2a_{3} = \frac{\gamma D}{{\alpha^{2} }}\sigma_{0} \left( {1 - \sigma_{0} } \right) - \mu, \hfill \\ a_{3} + a_{1} - a_{2} = \frac{1}{4} + \mu + \frac{\gamma D}{{\alpha^{2} }}\sigma_{0}^{2} . \hfill \\ \end{gathered}$$

Note that according to the NU method for a non-negative integer $n_{r} = 0, 1, 2,3, \ldots$, the hypergeometric type Eq. (30) has a unique polynomial $y = y_{{n_{r} }} (g)$ solution of degree $n_{r}$. On the other hand, according to Eq. (12), we have

39 $$\lambda_{{n_{r} }} = 2n_{r} \left[ {1 + \sqrt {a_{3} } + \sqrt {a_{3} + a_{1} - a_{2} } } \right] + n_{r} (n_{r} - 1).$$

In expressions (38) and (39), the left parts are equal, therefore, by equating the right parts of these expressions and solving the equation for $\sqrt {a_{3} }$, we find an analytical expression for the energy eigenvalues in the form:

40 $${M}^{2}-{E}^{2}=4{\alpha }^{2}{\left[\frac{p{\sigma }_{0}\left(1-{\sigma }_{0}\right)-\mu -\frac{1}{2}-{n}_{r}\left({n}_{r}+1\right)-\left(2{n}_{r}+1\right)q}{\left(2{n}_{r}+1\right)+2q}\right]}^{2}-\gamma D{\left(1-{\sigma }_{0}\right)}^{2},$$

where $p = \frac{\gamma D}{{\alpha^{2} }}\,,$ $q = \sqrt {\frac{1}{4} + \mu + p\sigma_{0}^{2} }$, the parameter $\mu$ is determined by the equation of the angular part of the KG equation.

Now we are trying to obtain the corresponding radial wave function $\chi (g)$ for the considered potential. $\sigma (g)$ and $\pi (g)$ from expressions (31) and (36) substituting into Eq. (8) and then solving the first order differential equation, we find the function $\varphi (g)$. She becomes equal

41 $$\varphi (g) = g^{{\sqrt {a_{3} } }} (1 - g)^{{\frac{1}{2} + \sqrt {a_{3} + a_{1} - a_{2} } }} .$$

The polynomial part of the wave function $\chi (g)$ is a polynomial $y_{{n_{r} }} (g)$ of hypergeometric type, which can be obtained from the Rodrigues relation (13). Solving Eq. (14) for the weight function $\rho (g)$, we find

42 $$\rho (g) = g^{{2\sqrt {a_{3} } }} (1 - g)^{{2\sqrt {a_{3} + a_{1} - a_{2} } }}.$$

Now substituting $\rho (g)$ and $\sigma (g)$ into Eq. (13), we get

43 $$y_{{n_{r} }} (g) = \frac{1}{{g^{{2\sqrt {a_{3} } }} (1 - g)^{{1 + 2\sqrt {a_{3} + a_{1} - a_{2} } }} }}\frac{{d^{{n_{r} }} }}{{dg^{{n_{r} }} }}\left[ {g^{{n_{r} + 2\sqrt {a_{3} } }} (1 - g)^{{n_{r} + 2\sqrt {a_{3} + a_{1} - a_{2} } }} } \right].$$

Let us now compare formula (43) with the definition of the Jacobi polynomials^78^

44 $$P_{{n_{r} }}^{(\alpha ,\beta )} (g) = \frac{{( - 1)^{{n_{r} }} }}{{2^{{n_{r} }} n_{r} !}}(1 - g)^{ - \alpha } (1 + g)^{ - \beta } \frac{{d^{{n_{r} }} }}{{dg^{{n_{r} }} }}\left[ {(1 - g)^{{\alpha + n_{r} }} (1 + g)^{{\beta + n_{r} }} } \right],$$

It follows from the results of this comparison that

45 $$y_{{n_{r} }} (g) = {\text{const}}\, \cdot P\,_{{n_{r} }}^{{(2\sqrt {a_{3} } ,2\sqrt {a_{3} + a_{1} - a_{2} } )}} (1 - 2g).$$

Substitution $\varphi_{{n_{r} }} (g)$ from Eq. (41) and $y_{{n_{r} }} (g)$ from Eq. (45) into Eq. (6) for the radial wave function gives the expression

46 $$\chi_{{n_{r} }} (g) = C_{{n_{r} }} g^{{\sqrt {a_{3} } }} (1 - g)^{{\frac{1}{2} + \sqrt {a_{3} + a_{1} - a_{2} } }} \cdot P_{{n_{r} }}^{{(2\sqrt {a_{3} } ,2\sqrt {a_{3} + a_{1} - a_{2} } )}} (1 - 2g).$$

The Jacobi polynomials are expressed in terms of the hypergeometric function as follows^78^

47 $$P_{{n_{r} }}^{(\alpha ,\beta )} (1 - 2g) = \frac{{\Gamma (n_{r} + \alpha + 1)}}{{n_{r} !\Gamma (\alpha + 1)}}{}_{2}F_{1} ( - n_{r} ,\alpha + \beta + n_{r} + 1,1 + \alpha ;g)$$

If to substitute expression (47) in (46) it is possible to express $\chi_{{n_{r} }} (g)$ radial wave function through hypergeometrical polynoms as:

48 $$\chi_{{n_{r} }} (g) = C_{{n_{r} }} g^{{\sqrt {a_{3} } }} (1 - g)^{K} \frac{{\Gamma (n_{r} + 2\sqrt {a_{3} } + 1)}}{{n_{r} !\Gamma (2\sqrt {a_{3} } + 1)}}{}_{2}F{}_{1}( - n_{r} ,2\sqrt {a_{3} } + 2K + n_{r} ,\,1 + 2\sqrt {a_{3} } ,g),$$

where $$K = \frac{1}{2} + q = \frac{1}{2} + \sqrt {\frac{1}{4} + \mu + p\sigma_{0}^{2} }$$.

The normalization constant $C_{{n_{r} }}$ in Eq. (48) is found from the condition of orthonormality of the radial wave function $\chi_{{n_{r} }} (g)$, as

49 $$\int\limits_{0}^{\infty } {\left| {\chi_{{n_{r} }} (r)} \right|^{2} dr = \frac{{1}}{2\alpha }} \int\limits_{0}^{1} {\left| {\chi_{{n_{r} }} \left( g \right)} \right|^{2} \frac{dg}{g}} = 1.$$

To calculate the integral, we use the following master formula^78^:50

As a result, we obtain the following expression for $C_{{n_{r} }}$

51 $$C_{{n_{r} }} = 2\sqrt {\frac{{\alpha \,\sqrt{a_3} \,n_{r} !(n_{r} + K + \sqrt{a_3} )\Gamma (n_{r} + 2\sqrt {a_{3} } + 2K)}}{{(n_{r} + K)\Gamma (n_{r} + 2K)\Gamma (n_{r} + 2\sqrt {a_{3} } + 1)}}} .$$

Here we take the potential parameters as $\sigma_{0} = 0.20$, $D = 2$, $\alpha = 0.30$, $\eta_{1} = 2$ and $\eta_{2} = 1$.

## Angular part of the Klein–Gordon equation

The eigenvalues and eigenvectors of the azimuthal part of the KG Eq. (23) can also be obtained by the NU method. To do this, we introduce a new variable $z = \cos \theta$. Its substitution into Eq. (23) gives

52 $$\Theta^{\prime\prime}(z) - \frac{2z}{{1 - z^{2} }}\Theta^{\prime}(\theta ) + \frac{1}{{(1 - z^{2} )^{2} }}[\mu (1 - z^{2} ) - m^{2} - \gamma (\eta_{1} + \eta_{2} z)]\Theta (z) = 0.$$

This equation is an equation of type (5) in which

53 $$\tilde{\tau }(z) = - 2z,\quad \sigma (z) = 1 - z^{2} ,\quad \tilde{\sigma }(z) = - \mu \,z^{2} - \gamma \eta_{2} z + (\mu - m^{2} - \gamma \eta_{1} )$$

Therefore, using the NU method, for the function $\pi (z)$ depending on the angle $\theta$, we find the following expression:

54 $$\pi (z) = \pm \sqrt {z^{2} (\mu - k) + \gamma \eta_{2} \,z - (\mu - \gamma \eta_{1} - m^{2} - k)} .$$

The constant parameter *k* takes the form:

55 $$k_{1,2} = \frac{{2\mu - m^{2} - \gamma \eta_{1} }}{2} \pm \frac{\vartheta }{2},$$

where $\vartheta = \sqrt {(m^{2} + \gamma \eta_{1} )^{2} - \gamma^{2} \eta_{2}^{2} }$. Corresponding function $\pi (z)$ for $k \equiv k_{2}$ defined as

56 $$\pi (z) = - z\sqrt {\frac{{m^{2} + \gamma \eta_{1} + \vartheta }}{2}} - \sqrt {\frac{{m^{2} + \gamma \eta_{1} - \vartheta }}{2}} .$$

From the condition of applicability of the NU method, it is known that the parameter $\tau (z)$ must satisfy the condition $\tau ^{\prime}(z) < 0$. Therefore, in this case $\tau (z)$ takes the form

57 $$\tau (z) = - 2z\left( {1 + \sqrt {\frac{{m^{2} + \gamma \eta_{1} + \vartheta }}{2}} } \right) - 2\sqrt {\frac{{m^{2} + \gamma \eta_{1} - \vartheta }}{2}} .$$

By introducing the notation $\xi = \sqrt {\frac{{m^{2} + \gamma \eta_{1} + \vartheta }}{2}}$, rewrite (56) and (57) in compact form

58 $$\pi (z) = - z\xi - \sqrt {\xi - \vartheta } ,\quad {\text{and}}\quad \tau (z) = - 2z\left( {1 + \xi } \right) - 2\sqrt {\xi^{2} - \vartheta } .$$

The parameter $\Lambda = k + \pi ^{\prime}(z)$ takes the form

59 $$\Lambda \equiv \Lambda_{N} = \mu - \xi^{2} - \xi = 2N\left( {1 + \xi } \right) + N(N - 1),$$

where $$N = 0,1,2, \ldots$$.

From here, for the parameter $\mu$ we obtain the expression

60 $$\mu = \xi^{2} + \xi + 2N\xi + N(N + 1) = (N + \xi )(N + \xi + 1).$$

If we put $\mu = l^{\prime}(l^{\prime} + 1)$ from here for $l^{\prime}$ we find

61 $$l^{\prime} = N + \xi .$$

At $\eta_{1} = \eta_{2} = 0$ we have $\xi = m$ and $l^{\prime} = l$, $N = l - m$.

Substituting expressions (59) into Eq. (38), we obtain the desired energy spectrum, expressed in terms of non-negative integer quantum numbers $n_{r} = 0,1,2, \ldots$ and $N = 0,1,2, \ldots$.

62 $$M^{2} - E^{2} = 4\alpha^{2} \left[ {\frac{{p\sigma_{0} (1 - \sigma_{0} ) - (N + \xi )(N + \xi + 1) - \frac{1}{2} - n_{r} (n_{r} + 1) - (2n_{r} + 1)q}}{{(2n_{r} + 1) + 2q}}} \right]^{2} - \gamma D(1 - \sigma_{0} )^{2} ,$$

where $$q = \sqrt {(N + \xi + 1/2)^{2} + p\sigma_{0}^{2} } .$$

Thus, by solving Eq. (60) to determine the energy spectrum, we can find the energy levels of the system for any values of the numbers $n_{r}$ and $N$.

Similarly to the radial wave function, we now find the explicit form of the wave function depending on the polar angle. First factorize the function $\Theta (z):$

63 $$\Theta (z) = \varphi (z)y(z).$$

Using Eq. (8), for $\varphi (z)$ obtain

64 $$\varphi (z) = (1 - z)^{{(K_{1} + K_{2} )/2}} \cdot (1 + z)^{{(K_{1} - K_{2} )/2}} ,$$

where

65 $$K_{1} = \sqrt {\frac{{m^{2} + \gamma \eta_{1} + \vartheta }}{2}} = \xi ,\quad K_{2} = \sqrt {\frac{{m^{2} + \gamma \eta_{1} - \vartheta }}{2}} = \sqrt {\xi^{2} - \vartheta } .$$

On the other hand, to find an explicit expression for polynomials $y_{{_{N} }} (z),\;N = 0,1,2, \ldots$ we first need to find the weight function $\rho (z)$. From the Pearson Eq. (14) for the weight function, we get:

66 $$\rho (z) = (1 - z)^{{K_{1} + K_{2} }} (1 + z)^{{K_{1} - K_{2} }} .$$

Accounting for (64) in formula (13) makes it possible to obtain the Rodrigues formula for the polynomial $y_{N} (z)$^76,78^:

67 $$y_{N} (z) = (1 - z)^{{ - (K_{1} + K_{2} )}} \cdot (1 + z)^{{K_{2} - K_{1} }} \frac{d}{dz}\left[ {(1 - z)^{{K_{1} + K_{2} + N}} \cdot (1 + z)^{{^{{K_{1} - K_{2} + N}} }} } \right].$$

Based on the definition (44) of Jacobi polynomials, we can write:

68 $$\frac{{d^{N} }}{{dz^{N} }}\left[ {\left( {1 - z} \right)^{{^{{K_{1} + K_{2} + N}} }} \cdot \left( {1 + z} \right)^{{^{{K_{1} - K_{2} + N}} }} } \right] = ( - 1)^{N} 2^{N} (1 - z)^{{K_{1} + K_{2} }} \cdot (1 + z)^{{^{{K_{1} - K_{2} }} }} P_{N}^{{(^{{K_{1} + K_{2} }} ,^{{K_{1} - K_{2} }} )}} (z).$$

Substituting expressions (64), (67) and (68) into Eq. (63), we obtain for $\Theta (z)$ the following expression:

69 $$\Theta_{N} (z) = \tilde{C}_{N} (1 - z)^{{(K_{1} + K_{2} )/2}} \cdot (1 + z)^{{(K_{1} - K_{2} )/2}} P_{N}^{{(K_{1} + K_{2} ,K_{1} - K_{2} )}} (z),$$

where $\tilde{C}_{N}$—normalization constant determined from the normalization condition. Using the orthogonality relation for Jacobi polynomials^78^:

70 $$\int\limits_{ - 1}^{1} {(1 - z)^{\mu } (1 + z)^{\nu } P_{m}^{(\mu ,\nu )} (z)P_{{n_{r} }}^{(\mu ,\nu )} (z)dz = \frac{{2^{\mu + \nu + 1} \Gamma (n_{r} + \mu + 1)\Gamma (n_{r} + \nu + 1)}}{{n_{r} !(2n_{r} + \mu + \nu + 1)\Gamma (n_{r} + \mu + \nu + 1)}}\delta_{mn} .}$$

after simple calculations of the normalization constant, we obtain:

71 $$\tilde{C}_{N} = \sqrt {\frac{{N!(2N + 2K_{1} + 1)\Gamma (N + 1)\Gamma (N + 2K_{1} + 1)}}{{2^{{2K_{1} + 1}} \Gamma (N + K{}_{1} + K_{2} + 1)\Gamma (N + K_{1} - K_{2} + 1)}}} .$$

Thus, after substituting Eqs. (21), (44) and (67) into Eq. (20), we directly obtain the total wave function for the considered quantum system with a combined potential defined as the sum of potentials of exponential and ring types

72 $$\begin{aligned} \psi_{{n_{r} N}} ({\mathbf{r}}) & = C_{{n_{r} N}} \frac{1}{r}e^{{ - 2\alpha \sqrt {a_{3} } r}} (1 - e^{ - 2\alpha r} )^{{\frac{1}{2} + \sqrt {a_{3} + a_{1} - a_{2} } }} P_{{n_{r} }}^{{(2\sqrt {a_{3} } ,2\sqrt {a_{3} + a_{1} - a_{2} } )}} (1 - 2e^{ - 2\alpha r} ) \\ & \quad \cdot (1 - \cos \theta )^{{(K_{1} + K_{2} )/2}} (1 + \cos \theta )^{{(K_{1} - K_{2} )/2}} \cdot P_{N}^{{(K_{1} + K_{2} ,K_{1} - K_{2} )}} (\cos \theta ). \\ \end{aligned}$$

Wave function (72) satisfies the normalization condition

73 $$\int {\left| {\psi ({\mathbf{r}})} \right|^{2} d\,{\mathbf{r}} = 1}$$

with $$C_{{n_{r} N}} = C_{{n_{r} }} \cdot \tilde{C}_{N}$$.

## Limiting case

In this section, we find the nonrelativistic limits of the radial wave function (46) and energy Eq. (62) of the system under consideration and compare them with the corresponding results in^52^. In doing so, we take into account that the Eq. (17) reduces to the Schrödinger equation for the potential 2 $V({\mathbf{r}})$. First, we restore the constants $\hbar$ and $c$ in formulas (46) and (62), i.e. we will make replacements in them

74 $$\varepsilon^{2} \to \frac{{E^{2} - M^{2} c^{4} }}{{\hbar^{2} c^{2} }},\quad \gamma \to \frac{{2\left( {E + Mc^{2} } \right)}}{{\hbar^{2} c^{2} }}$$

and give the nonrelativistic limits of the parameters ${a}_{1}, {a}_{2}, {a}_{3}$ and the energy *E*:

75 $$\begin{aligned} & \mathop {\lim }\limits_{c \to \infty } a_{1} = a_{1N} = \frac{1}{4} + \lambda^{2} + k\left( {1 + \sigma_{0} } \right)^{2} , \\ & \mathop {\lim }\limits_{c \to \infty } a_{2} = a_{2N} = 2\lambda^{2} + 2k\left( {1 - \sigma_{0}^{2} } \right) - \mu_{N} , \\ & \mathop {\lim }\limits_{c \to \infty } a_{3} = a_{3N} = \lambda^{2} + k\left( {1 - \sigma_{0} } \right)^{2} , \\ & \mathop {\lim }\limits_{c \to \infty } \left( {E - Mc^{2} } \right) = E_{N} > 0. \\ \end{aligned}$$

Here we use the following notation (compare with^52^):

76 $$\lambda = \sqrt { - \frac{{ME_{N} }}{{2\alpha^{2} \hbar^{2} }}} ,\quad k = \frac{MD}{{\alpha^{2} \hbar^{2} }},\quad \mu_{N} = l_{N}{\prime} \left( {l_{N}{\prime} + 1} \right),$$

where ${\mu }_{N}$ obtained from $\mu$ by replacing $\gamma$ with $\gamma_{N} = 4M/\hbar^{2} .$

Now it is easy to calculate the nonrelativistic limit of function (46), which has the form $\lim_{c \to \infty } = \chi_{{n_{r} }} = \chi_{{n_{r} N}}$, where

77 $$\chi_{n,N} = N_{{n_{r} }} g^{{\sqrt {a_{3N} } ,}} (1 - g)^{{\frac{1}{2} + \sqrt {a_{3N} + a_{1N} - a_{2} N} }} P_{{n_{r} }}^{{\left( {2\sqrt {a_{3N} } ,2\sqrt {a_{3N} + a_{1N} - a_{2N} } } \right)}} (1 - 2g).$$

The calculation of the nonrelativistic limit of the energy equation is also not difficult, and as a result we get

78 $$E_{{n_{r} N}} = - \frac{{2\alpha^{2} \hbar^{2} }}{M}\left\{ {\left[ {\frac{{\left( {n_{r} + 1} \right)^{2} - 4k\sigma_{0} \left( {1 - \sigma_{0} } \right) + l_{N}{\prime} \left( {l_{N}{\prime} + 1} \right) + \left( {2n_{r} + 1} \right)\delta }}{{2\left( {n_{r} + \delta + 1} \right)}}} \right]^{2} - k\left( {1 - \sigma_{0} } \right)^{2} } \right\}.$$

Thus, expressions (77) and (78) are nonrelativistic wave functions and energy spectrum of the non-central potential $V\left( r \right) = D\left[ {1 - \sigma_{0} \coth (\alpha r)} \right]^{2} + (\eta_{1} + \eta_{2} \cos \theta )/r^{2} \sin^{2} \theta$ for the arbitrary ${l}{\prime}$-wave, where $l^{\prime} = N + \xi$.

It is easy to check that for $\eta_{1} = \eta_{2} = 0$ formulas (77) and (78) coincide with the corresponding formulas in^53^ with normalization constant:

79 $${\mathcal{N}} = \sqrt {\frac{{2\alpha \left( {n_{r} + \delta + \beta + 1} \right)\Gamma \left( {n_{r} + 2\beta + 1} \right)\Gamma \left( {n_{r} + 2\delta + 2\beta + 2} \right)}}{{n_{r} !\left( {n_{r} + \delta + 1} \right)\Gamma \left( {n_{r} + 2\delta + 2} \right)\Gamma \left( {2\beta } \right)\Gamma \left( {2\beta + 1} \right)}}} .$$

## Discussion of the numerical results

In this paper, energy eigenvalues and their corresponding eigenfunctions are obtained for arbitrary states $l \ne 0$ by analytically solving the modified Klein–Gordon equation for the sum of the exponential and ring-shaped potential applying the Nikiforov–Uvarov method.

In this section, we carry out numerical evaluations of our analytical results. We carry out the numerical calculations using the MATLAB R2023a and MATHEMATICA 8 package programs. The figures in this work have been drawn with help of the program ORIGIN 8.5.1.

**Figure 3:**
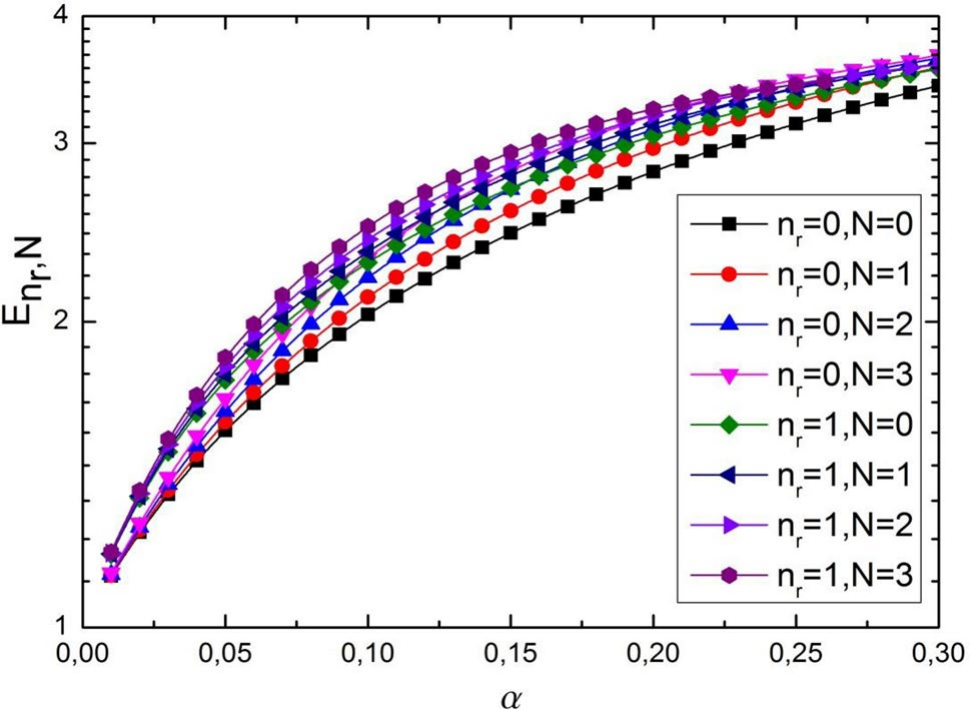
The dependence energy spectrum as a function of $\alpha$ for different values of $n_{r} = 0,1$ and $N = 0,1,2,3,$ where we used $\eta_{1} = \eta_{2} = 1$, $D = 4$, $\sigma_{0} = 0.20$, $M = 1$, $m = 0$.

**Figure 4:**
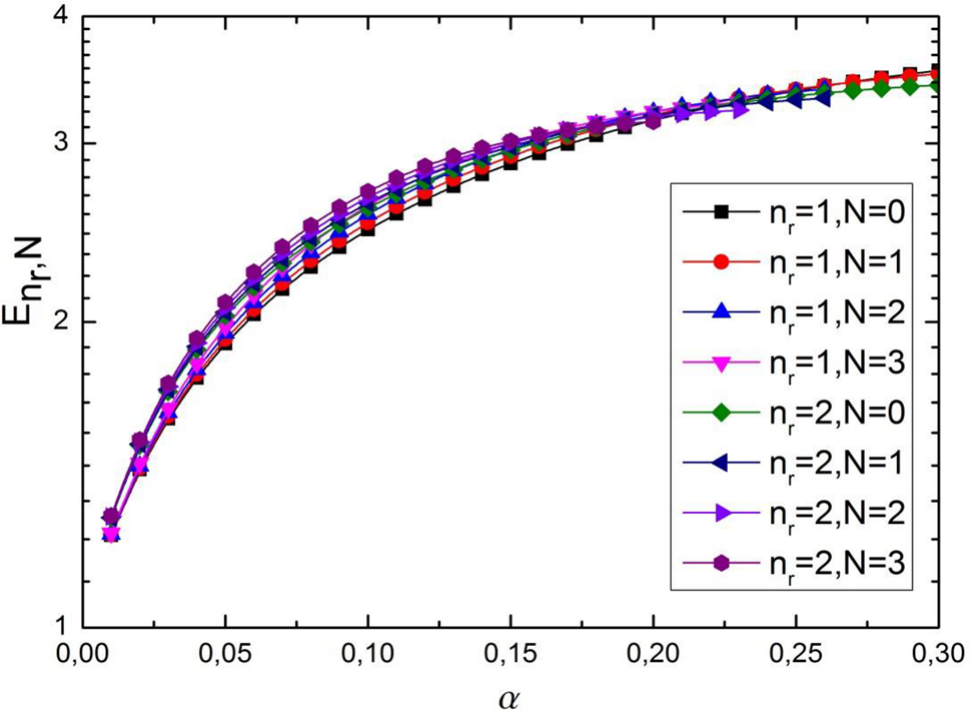
The dependence energy spectrum as a function of $\alpha$ for different values of $n_{r} = 1,2$ and $N = 0,1,2,3,$, where we used $\eta_{1} = \eta_{2} = 1$, $D = 4$ and $\sigma_{0} = 0.2$, $M = 1$, $m = 0$.

In Tables 1 and 2, we present several energy levels $E_{{n_{r} N}}$ the dependence of the screening parameter for few values of the radial $n_{r}$ and $N$ quantum numbers. In numerical estimation the energy eigenvalues of the screening parameter $\alpha$ we used the parameters of the potential as $\eta_{1} = \eta_{2} = 1$, $D = 4$ and $\sigma_{0} = 0.2$, $M = 1$, $m = 0$. These parameters used in the numerical calculations are taken arbitrarily for illustrative purposes. In Figs. 3 and 4 are visualized the dependence energy spectrum of the screening parameter $\alpha$ for different radial $n_{r}$ and $N = 0,1,2,3$ quantum numbers. Here, $n_{r}$ is the number of nodes of radial wave functions, and the usual principal quantum number is given by $n = n_{r} + l^{\prime} + 1$. The screening parameter is changed in the interval $\alpha \in [0.01 \div 0.30]$. It is also from the Figs. 3 and 4 seen that the energy spectrum are monotonically increasing with an increase in the screening parameter. Such an increase is almost logarithmic with increasing screening parameter $\alpha$. Moreover, the energy eigenvalues increase with the increment as $n_{r}$ and $N$ . Should be noted that states with the same total value $n_{r} + N$ have close energy values to each other. For example, the following states ($n_{r}$,$N$) = (0, 1), (1, 0), ($n_{r}$,$N$) = (0, 2), (1, 1), (2, 0). In addition, it was shown that the energy eigenvalues and the corresponding wave eigenfunctions are sensitive to the choice of radial $n_{r}$, orbital *l* and $N$ quantum numbers. Therefore, the study of the analytical solution of the modified KG equation for a linear combination of exponential potential and ring-shaped potential in the framework of quantum mechanics can contribute to obtaining valuable information about the dynamics in nuclear, atomic and molecular physics and open a new window for deeper research. Must be noted that central potential describe quantum system in the radial direction (region), but noncentral potential is describe quantum system in the azimuthal region. Thus, the combined (noncentral) potential keep a more information regarding the central potential, and give us for getting the better results about quantum mechanical system.

**Table 1:** Bound state energy eigenvalues $E_{{n_{r} N}}$ as a function screening parameter $\alpha$ for various values of $n_{r}$ and $N$.

$\alpha$	$n_{r}$ = 0, N = 0	$n_{r}$ = 0, N = 1	$n_{r}$ = 0, N = 2	$n_{r}$ = 0, N = 3	$n_{r}$ = 1, N = 0	$n_{r}$ = 1, N = 1	$n_{r}$ = 1, N = 2	$n_{r}$ = 1, N = 3
	$$E_{{n_{r} N}}$$							
0.01	1.1243	1.1255	1.1279	1.1316	1.1804	1.1816	1.1841	1.1871
0.02	1.2415	1.2476	1.2561	1.2671	1.3416	1.3477	1.3550	1.3647
0.03	1.3525	1.3660	1.3837	1.4056	1.4893	1.5002	1.5149	1.5338
0.04	1.4600	1.4813	1.5094	1.5442	1.6254	1.6431	1.6656	1.6937
0.05	1.5631	1.5936	1.6333	1.6809	1.7523	1.7767	1.8079	1.8457
0.06	1.6626	1.7035	1.7554	1.8152	1.8719	1.9031	1.9427	1.9891
0.07	1.7596	1.8103	1.8744	1.9464	1.9843	2.0221	2.0697	2.1246
0.08	1.8536	1.9153	1.9904	2.0728	2.0911	2.1356	2.1906	2.2516
0.09	1.9446	2.0166	2.1027	2.1948	2.1918	2.2430	2.3047	2.3712
0.10	2.0337	2.1161	2.2119	2.3117	2.2882	2.3453	2.4121	2.4835
0.11	2.1204	2.2125	2.3169	2.4237	2.3804	2.4426	2.5140	2.5879
0.12	2.2046	2.3065	2.4188	2.5299	2.4680	2.5351	2.6105	2.6855
0.13	2.2873	2.3981	2.5168	2.6312	2.5519	2.6233	2.7008	2.7762
0.14	2.3679	2.4866	2.6108	2.7271	2.6324	2.7069	2.7863	2.8601
0.15	2.4463	2.5726	2.7008	2.8174	2.7094	2.7869	2.8665	2.9382
0.16	2.5232	2.6559	2.7875	2.9028	2.7832	2.8625	2.9419	3.0096
0.17	2.5980	2.7368	2.8699	2.9834	2.8537	2.9349	3.0124	3.0756
0.18	2.6709	2.8149	2.9489	3.0588	2.9218	3.0035	3.0786	3.1360
0.19	2.7423	2.8903	3.0243	3.1293	2.9868	3.0685	3.1403	3.1915
0.20	2.8119	2.9633	3.0957	3.1958	3.0493	3.1305	3.1982	3.2416
0.21	2.8796	3.0338	3.1641	3.2574	3.1091	3.1891	3.2520	3.2867
0.22	2.9462	3.1015	3.2288	3.3148	3.1665	3.2446	3.3017	3.3276
0.23	3.0106	3.1671	3.2901	3.3685	3.2220	3.2971	3.3481	3.3643
0.24	3.0731	3.2300	3.3484	3.4183	3.2748	3.3469	3.3908	3.3966
0.25	3.1348	3.2907	3.4036	3.4644	3.3258	3.3939	3.4302	3.4253
0.26	3.1943	3.3490	3.4558	3.5068	3.3743	3.4381	3.4665	3.4500
0.27	3.2523	3.4052	3.5052	3.5458	3.4210	3.4802	3.4998	3.4728
0.28	3.3087	3.4592	3.5519	3.5818	3.4659	3.5196	3.5303	3.4985
029	3.3636	3.5107	3.5959	3.6145	3.5089	3.5568	3.5577	3.5194
0.30	3.4174	3.5605	3.6374	3.6239	3.5501	3.5916	3.5828	3.5216

For potential parameters used $\eta_{1} = \eta_{2} = 1$, $D = 4$ and $\sigma_{0} = 0.2$, $M = 1$, $m = 0$, ($n_{r}$; $N$) = (0, 1; 0, 1, 2, 3).

**Table 2:** Bound state energy eigenvalues $E_{{n_{r} N}}$ as a function screening parameter $\alpha$ for various values of $n_{r}$ and $N$.

$\alpha$	$n_{r}$ = 2, N = 0	$n_{r}$ = 2, N = 1	$n_{r}$ = 2, N = 2	$n_{r}$ = 2, N = 3	$n_{r}$ = 3, N = 0	$n_{r}$ = 3, N = 1	$n_{r}$ = 3, N = 2	$n_{r}$ = 3, N = 3
	$$E_{{n_{r} N}}$$							
0.01	1.2329	1.2347	1.2366	1.2396	1.2830	1.2842	1.2866	1.2891
0.02	1.4319	1.4368	1.4435	1.4526	1.5131	1.5173	1.5234	1.5314
0.03	1.6064	1.6162	1.6284	1.6449	1.7090	1.7169	1.7279	1.7419
0.04	1.7627	1.7773	1.7963	1.8201	1.8793	1.8915	1.9073	1.9275
0.05	1.9049	1.9244	1.9501	1.9806	2.0300	2.0459	2.0673	2.0923
0.06	2.0349	2.0593	2.0911	2.1277	2.1652	2.1851	2.2101	2.2400
0.07	2.1552	2.1838	2.2211	2.2632	2.2870	2.3102	2.3389	2.3718
0.08	2.2662	2.2998	2.3407	2.3874	2.3981	2.4234	2.4548	2.4902
0.09	2.3694	2.4066	2.4518	2.5012	2.4991	2.5266	2.5598	2.5958
0.10	2.4661	2.5067	2.5543	2.6056	2.5916	2.6202	2.6544	2.6901
0.11	2.5565	2.5995	2.6495	2.7005	2.6764	2.7060	2.7399	2.7737
0.12	2.6410	2.6865	2.7368	2.7869	2.7542	2.7838	2.8168	2.8479
0.13	2.7209	2.7673	2.8177	2.8656	2.8259	2.8552	2.8857	2.9126
0.14	2.7954	2.8430	2.8918	2.9364	2.8918	2.9199	2.9471	2.9688
0.15	2.8659	2.9132	2.9602	2.9999	2.9529	2.9785	3.0017	3.0167
0.16	2.9321	2.9788	3.0231	3.0569	3.0084	3.0316	3.0499	3.0573
0.17	2.9944	3.0399	3.0801	3.1073	3.0600	3.0798	3.0920	3.0908
0.18	3.0536	3.0966	3.1323	3.1519	3.1073	3.1232	3.1287	3.1171
0.19	3.1088	3.1494	3.1793	3.1903	3.1506	3.1616	3.1595	3.1372
0.20	3.1610	3.1982	3.2220	3.2233	3.1903	3.1958	3.1854	3.1512
0.21	3.2101	3.2434	3.2599	3.2507	3.2263	3.2260	3.2062	3.1648
0.22	3.2562	3.2852	3.2941	3.2736	3.2593	3.2523	3.2220	3.2158
0.23	3.2999	3.3237	3.3240	3.2916	3.2892	3.2748	3.2336	3.2938
0.24	3.3408	3.3591	3.3502	3.3123	3.3160	3.2938	3.3327	3.3258
0.25	3.3792	3.3911	3.3725	3.3208	3.3405	3.3093	3.3398	3.3328
0.26	3.4152	3.4204	3.3917	3.3269	3.3618	3.3215	3.3416	3.3496
0.27	3.4491	3.4473	3.4102	3.3318	3.3807	3.3279	3.3489	3.3529
0.28	3.4808	3.4711	3.4199	3.3389	3.3972	3.3328	3.3528	3.3688
029	3.5101	3.4924	3.4278	3.3405	3.4113	3.3429	3.3615	3.3738
0.30	3.5379	3.5117	3.4407	3.3486	3.4232	3.3527	3.3689	3.3837

For potential parameters used $\eta_{1} = \eta_{2} = 1$, $D = 4$ and $\sigma_{0} = 0.2$, $M = 1$, $m = 0$, ($n_{r}$; $N$) = (2, 3; 0, 1, 2, 3).

Finally, the results obtained are of interest not only to a theoretical physicist, but also to an experimental physicist, since they are accurate and more general.

## Conclusion

In this study, we presented solutions of the KG equation in the bound state with a new combined exponential type potential plus ring-shaped potential, using an approximation scheme to work with the centrifugal term. For an analytical solution in the framework of ordinary quantum mechanics, the well-established method is used. Equations for the numerical calculation of the energy eigenvalues and the corresponding normalized wave functions of the specified quantum system for any $n_{r}$ and $N$ are obtained analytically, and they are presented in a closed and compact form. The normalized wave functions are expressed in terms of the hypergeometric function and of the Jacobi polynomial. It is obvious that the solutions in the bound state are more stable for the potential we are considering than individual cases. We have estimated the change in energy eigenvalues for various parameters and quantum numbers. The results obtained for the energy spectrum show that the energy eigenvalues are sensitive to the screening parameter $\alpha$ as well as the quantum numbers $n_{r}$ and $N$.

Therefore, obtaining results can provide important information about the dynamics in atomic and molecular physics and makes it possible to study this problem in more depth. Thus, we can conclude that the results obtained by us will be of interest not only to a theoretical physicist, but also to an experimental physicist, due to accurate and more general results.
